# CXCR4/CXCL12 axis counteracts hematopoietic stem cell exhaustion through selective protection against oxidative stress

**DOI:** 10.1038/srep37827

**Published:** 2016-11-25

**Authors:** Yanyan Zhang, Mallorie Dépond, Liang He, Adlen Foudi, Edward Owusu Kwarteng, Evelyne Lauret, Isabelle Plo, Christophe Desterke, Philippe Dessen, Nobutaka Fujii, Paule Opolon, Olivier Herault, Eric Solary, William Vainchenker, Virginie Joulin, Fawzia Louache, Monika Wittner

**Affiliations:** 1 Paris-Saclay University, UMRS-1170, Gustave Roussy, Villejuif, France; 2 Paris-Saclay University, INSERM U935, Andre Lwoff Institute, Paul Brousse Hospital, Villejuif, France; 3 Paris Descartes University, CNRS (UMR 8104), Inserm U1016, Institut Cochin, Paris, France; 4 Paris-Saclay University, UFR Medicine, INSERM UMS 33, Andre Lwoff Institute, Paul Brousse Hospital, Villejuif, France; 5 Bioinformatic platform, UMS AMMICA, INSERM US23/CNRS UMS3665, Gustave Roussy, Villejuif, France; 6 Kyoto University, Graduate School of Pharmaceutical Sciences, Kyoto, Japan; 7 Laboratoire de pathologie expérimentale, Gustave Roussy, Villejuif, France; 8 CNRS UMR 7292 GICC, Tours, France; 9 CNRS GDR 3697 MicroNiT, France.

## Abstract

Hematopoietic stem cells (HSCs) undergo self-renewal to maintain hematopoietic homeostasis for lifetime, which is regulated by the bone marrow (BM) microenvironment. The chemokine receptor CXCR4 and its ligand CXCL12 are critical factors supporting quiescence and BM retention of HSCs. Here, we report an unknown function of CXCR4/CXCL12 axis in the protection of HSCs against oxidative stress. Disruption of CXCR4 receptor in mice leads to increased endogenous production of reactive oxygen species (ROS), resulting in p38 MAPK activation, increased DNA double-strand breaks and apoptosis leading to marked reduction in HSC repopulating potential. Increased ROS levels are directly responsible for exhaustion of the HSC pool and are not linked to loss of quiescence of CXCR4-deficient HSCs. Furthermore, we report that CXCL12 has a direct rescue effect on oxidative stress-induced HSC damage at the mitochondrial level. These data highlight the importance of CXCR4/CXCL12 axis in the regulation of lifespan of HSCs by limiting ROS generation and genotoxic stress.

Reactive oxygen species (ROS) are produced during oxidative respiration or through exogenous environmental stresses, such as ionizing radiations or genotoxic treatments. Physiological concentrations of ROS play a role in signal transduction^1^,^2^, but at high concentrations they can oxidize cell constituents leading to protein carbonylation, lipid peroxidation and DNA damage that activate multiple apoptosis pathways^3^.

By far, the most important source for ROS is mitochondria^4^,^5^ and their endogenous production as by-products of aerobic respiration is thought to be the cause of most oxidative damages observed in mammals and particularly during aging^6^,^7^.

To avoid accumulation of oxidative stress, cells have evolved mechanisms to fine-tune ROS levels. They involve distinct groups of specialized proteins such as superoxide dismutase (SOD), catalase and glutathione peroxidase. Reduced glutathione (GSH), which also exists in the cell in its oxidized form (GSSG), is considered as the most abundant molecule among endogenous antioxidants. Alteration in its redox status serves as an indicator of oxidative stress when antioxidant defense mechanisms are not completely efficient and is a common feature of ageing and many pathological situations including AIDS, neurodegenerative diseases and cancer.

Hematopoietic stem cells (HSCs) are defined as cells capable of both self-renewal and differentiation into any of the hematopoietic cell lineages, properties that allow hematopoietic reconstitution^8^,^9^. Long-term maintenance of HSCs is precisely regulated by the equilibrium between proliferation and quiescence to maintain their numbers and lifespan. Defects in these processes lead to hematopoietic insufficiencies and to the development of hematopoietic malignancies.

A low level of ROS is a hallmark of primitive HSCs, and moderate, physiological elevation in ROS levels in these cells enhances motility, short-term repopulation and repair processes^10^,^11^. However, when ROS are excessively elevated, they lead to irreversible damage, such as senescence and apoptosis resulting in premature exhaustion of HSC self-renewal. In line with this, disruption of oxidative stress-regulating pathways in *Atm*^−/−^ mice leads to premature HSC exhaustion due to loss of quiescence and decline in their self-renewal capacity^12^. Moreover, it has been reported that the antioxidative enzyme GPx-3 is a determinant of self-renewal of HSCs^13^. Increased ROS levels, as a result of mitochondrial dysfunction^14^,^15^ or achieved by deletion of antioxidant stress genes such as FOXOs^16^ or BMI^17^, have been shown to influence HSC differentiation and long-term maintenance. As ROS elevation results in DNA damage, they may contribute to genomic instability^18^ and accumulation of mutations and deletion that may lead to cancer^19^. Conversely, extremely low ROS levels result in defects in differentiation and repopulation capacity^20^. Thus, ‘balanced ROS levels’ appear to be essential for long-term functions of HSCs. However, the molecular mechanisms that regulate ROS levels and oxidative stress responses are poorly understood.

HSCs are localized in a microenvironment known as the stem cell “niche,” where they are maintained in an undifferentiated and quiescent state by various types of niche-related factors^21^,^22^,^23^. Within the niches, the chemokine stromal cell-derived factor-1 (SDF-1, also termed CXCL12) and its major receptor *CXCR4* expressed on HSCs are key factors. *CXCR4*/CXCL12 signaling influences many aspects of HSPC biology including migration, retention within stem cell niches, proliferation and quiescence^24^,^25^,^26^,^27^,^28^. Nonetheless, its requirement for HSPC biology remains to be explored. Indeed, significant discrepancies were reported on the effects of *CXCR4* deletion regarding the long-term maintenance of the HSC pool using inducible mouse models. *CXCR4* deletion achieved with poly(I)-poly(C)-inducible Cre-transgenic mice, resulted in sharp deleterious effects on HSCs, whereas *CXCR4*^−/−^ HSC numbers were maintained in tamoxifen-inducible Cre-transgenic mice^28^,^29^.

Here, we uncovered a specific function for CXCR4/CXCL12 axis in the prevention of ROS elevation, apoptosis and DNA damage in HSCs and provide missing links between CXCR4/CXCL12 pathway and ROS generation.

## Results

### Disruption of CXCR4 receptor leads to progressive exhaustion of the adult BM HSC pool in CXCR4^−/−^ chimeras

**Figure 1:**
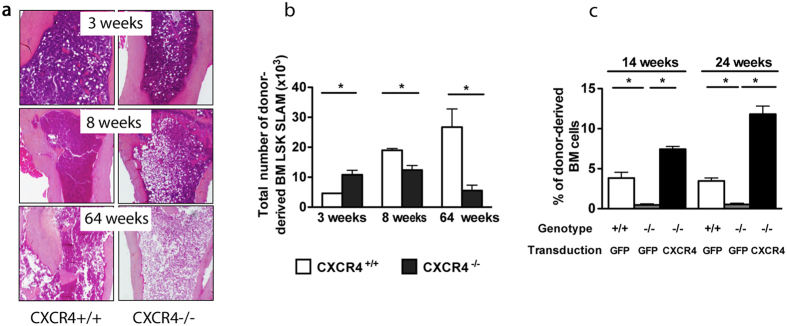
Defective hematopoiesis in BM of *CXCR4*^−/−^ chimeras. (**a**) BM sections from CXCR4^+/+^ and CXCR4^−/−^ chimeras were stained with hematoxylin/eosin/safran. Chimeric mice were analyzed 3, 8 and 64 weeks after engraftment of eight-week-old, lethally irradiated mice with 5 × 10^6^ CXCR4^+/+^ or CXCR4^−/−^ FL cells. (**b**) Total donor-derived LSK-SLAM cell numbers in BM. Mean ± SEM from 3 independent experiments (5–7 mice per group). (**c**) Restored hematopoietic reconstitution potential by retroviral expression of CXCR4 in CXCR4^−/−^ HSCs. Mean ± SEM of the percentages of donor-derived BM cells 14 and 24 weeks post engraftment. Data represent a pool from 8 mice over two independent experiments.

Several studies have demonstrated that some chemical agents including tamoxifen or Poly(I)-Poly(C) used in conditional knockout experiments to induce gene deletion, may not be totally harmless to HSCs by direct interference with their proliferative state and ROS levels^30^,^31^. Therefore, to avoid any caveats owing to chemical inducers, we chose to carry out this study by transplantation experiments using conventional CXCR4 KO fetal liver (FL) cells in which CXCR4 is constitutively inactive. Of note, it has been demonstrated that FL HSCs can reach an adult state between 4 to 5 weeks post transplant^32^. Using this approach, we have previously demonstrated that CXCR4^−/−^ FL cells have kept the potential to reconstitute hematopoiesis in lethally irradiated mice, albeit in the short term^26^. To address whether CXCR4^−/−^ FL cells contribute to long-term hematopoiesis, we analyzed in more depth the HSPC compartment of chimeric mice reconstituted either with CD45.2^+^ CXCR4^−/−^ or CXCR4^+/+^ FL cells over time. The origin of hematopoietic cells in the BM, spleen and peripheral blood (PB) of these chimeric mice was evaluated by dual labeling with antibodies against CD45.1 (host) and CD45.2 (donor). CXCR4^−/−^ chimeric mice displayed a progressive yet marked decrease in BM cellularity over time in comparison to CXCR4^+/+^ chimeras (Fig. 1a). This decrease was noticeable starting at 8 weeks post-transplantation and was profound after one year of reconstitution (Fig. 1a). Concomitantly, after an initial increase (3 weeks), the number of CXCR4^−/−^ BM enriched long-term HSCs (LSK-SLAM: Lin^−^Sca-1^+^c-Kit^+^CD150^+^CD48^−^CD41^−^) significantly decreased over time (Fig. 1b). This BM HSC exhaustion was accompanied by a long-term deficit in BM progenitor content (Supplementary Figure S1a) and an early and sustained increase in splenic HSPC-enriched LSK (Lin^−^Sca-1^+^c-Kit^+^) and colony forming cells (CFC) (Supplementary Figure S1b, S1c). Notably, not only did we observe a dramatic increase in the number of donor-derived LSK cells in the peripheral blood (PB) of CXCR4^−/−^ chimeras, as has been reported before^24^,^26^, but also the presence of LSK-SLAM cells was observed (Supplementary Figure S1d, S1e). Furthermore, to determine whether the BM of CXCR4^−/−^ chimeric mice contained genuine LT-HSCs capable of long-term reconstitution, we performed rescue experiments. Three months after transplantation, donor-derived BM cells from CXCR4^−/−^ chimeras were transduced with retrovirus encoding full length CXCR4 or GFP and injected into secondary recipients. As shown in Fig. 1c, CXCR4^−/−^ cells displayed a marked decrease in their hematopoietic repopulating capacities in comparison to CXCR4^+/+^ cells (^−/−^GFP vs. ^+/+^GFP). This defect was, however, totally rescued by the re-expression of CXCR4 (^−/−^GFP vs. ^−/−^CXCR4). In addition, the reconstitution fitness of ^−/−^CXCR4 cells was even higher than controls (^−/−^GFP vs. ^+/+^CXCR4). These results demonstrate that CXCR4^−/−^ HSCs from chimeric mice exhibit a cell autonomous long-term reconstitution defect that can be rescued by expression of CXCR4.

### HSPCs of CXCR4^−/−^ chimeras exhibit increased oxidative stress

**Figure 2:**
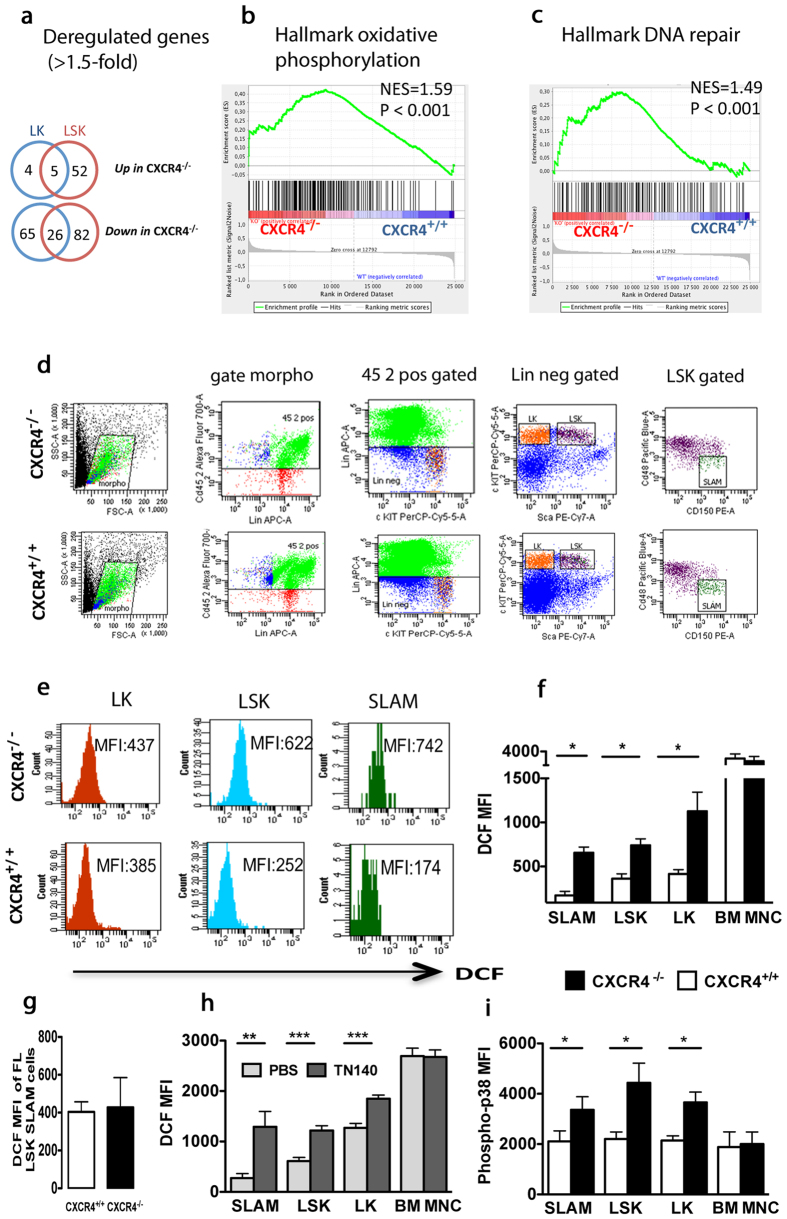
Increased endogenous ROS production in BM of CXCR4^−/−^ chimeras. (**a**) Whole genome microarray analysis of CXCR4^−/−^ and CXCR4^+/+^ LK and LSK cells. Venn diagrams show number of differentially expressed genes that are at least 1.5-fold changed. (**b**,**c**) GSEA of CXCR4^−/−^ and CXCR4^+/+^ LSK cells showing significant enrichment of Hallmark Oxidative phosphorylation (**b**) and Hallmark DNA Repair (**c**) gene sets in CXCR4^−/−^ cells. (**d**) Full gating strategy with actual staining plots for immunophenotypic analysis of HSCs from CXCR4^+/+^ and CXCR4^−/−^ chimeras performed 10 weeks after FL cell transplantation. (**e**) Mean Fluorescence Intensity (MFI) of DCF on BM progenitor cells from CXCR4^+/+^ and CXCR4^−/−^ chimeras according to the gating strategy described in (**d**). DCF staining showed increased intracellular ROS in CXCR4^−/−^ LT-HSCs and HSPCs. Data are representative of 6 independent experiments. (**f**) DCF MFI of donor-derived LSK-SLAM (SLAM), LSK, LK and BM mononuclear (BM MNC) cells. Mean ± SEM from 6 mice per group, pooled from 2 experiments. (**g**) DCF MFI of LSK-SLAM cells from CXCR4^+/+^ and CXCR4^−/−^ FL cells. Data from 3 independent experiments are shown. (**h**) Increased intracellular ROS in ten-week-old WT mice treated with CXCR4 antagonist TN140. Mean DCF MFI ± SEM from 6 mice per experiment in 2 independent experiments. (**i**) Increased phosphorylation of p38 MAPK in CXCR4^−/−^ HSPCs. Mean phospho-p38 MFI ± SEM in donor-derived LSK-SLAM, LSK, LK and BM mononuclear (BM-MNC) fractions. Data from 8 mice per group over 2 independent experiments are shown. CXCR4^+/+^ and CXCR4^−/−^ cells were obtained from chimeric mice 10 weeks after FL cell transplantation.

To shed light on the molecular pathways regulated by CXCR4/CXCL12 axis, we assessed genome-wide mRNA expression in CXCR4^−/−^ and CXCR4^+/+^ HSPCs sorted from chimeric mice. Since we failed to isolate sufficient numbers of CXCR4^−/−^ LSK-SLAM cells, we analyzed LSK and myeloid progenitors (LK, Lin^−^Sca-1^−^c-Kit^+^) cells. Significance Analysis of Microarray (SAM; pubmed#11309499) identified 165 and 100 differentially expressed genes in LSK and LK cells, respectively (>1.5-fold change; FDR <5%) (Fig. 2a). Gene Set Enrichment Analysis (GSEA; MsigDB 5.1) revealed that hallmarks of oxidative phosphorylation and DNA repair were significantly enriched in CXCR4^−/−^ LSK cells (Fig. 2b,c). Using 5-(and 6)-chloromethyl-2′-7′-dichlorofluorescein-diacetate (CM-H_2_DCF DA) staining and immunophenotypic analysis of BM progenitor cells from CXCR4^+/+^ and CXCR4^−/−^ chimeras according to the gating strategy described in Fig. 2d, we confirmed elevated ROS levels in CXCR4^−/−^ HSPCs. As shown in Fig. 2e,f, ROS levels were significantly increased in LK, LSK and LSK-SLAM from CXCR4^−/−^ BM compared to CXCR4^+/+^ BM cells. In contrast, no differences were observed in mature BM cells (Fig. 2f) and in LSK-SLAM cells directly obtained from FL of CXCR4^+/+^ and CXCR4^−/−^ embryos (Fig. 2g). To study whether CXCR4 functions on ROS regulation could be recapitulated by another strategy of inhibition, we evaluated the effects of CXCR4 antagonist 4F-benzoyl-TN14003 (TN140) on ROS levels in ten-week-old wild type mice. Similarly to CXCR4^−/−^ cells, LSK-SLAM, LSK and LK cells from wild type treated mice showed increased endogenous ROS levels (Fig. 2h). Thus, CXCR4 receptor disruption results in increased oxidative stress, specifically encountered in HSPCs. Stress-activated p38 MAPK is involved in different situations including oxidative stress^12^. Flow cytometry analysis of CXCR4^+/+^ and CXCR4^−/−^ BM at 8 weeks after transplantation revealed that phosphorylation of p38 MAPK was significantly enhanced in LSK-SLAM, LSK and LK cells of BM from CXCR4^−/−^ chimeras compared to CXCR4^+/+^ counterparts (Fig. 2i). No activation of the p38 MAPK pathway was observed in differentiated BM cell populations of CXCR4^−/−^ chimeras (Fig. 2i).

### *In vivo* treatment with ROS scavenger N-acetyl-L-cysteine prevents apoptosis and DNA double-strand breaks in HSPCs from CXCR4^−/−^ chimeras

**Figure 3:**
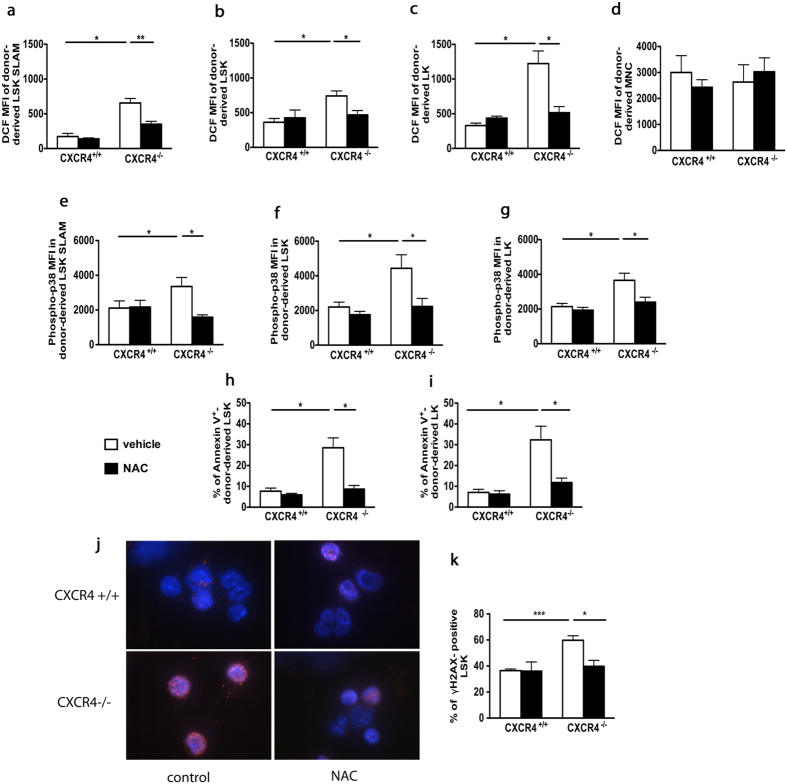
Defects in *CXCR4*^−/−^ HSPC function are counteracted by the antioxidant NAC. (**a**–**d**) DCF MFIs of donor-derived BM subsets of *CXCR4*^+/+^ and *CXCR4*^−/−^ chimeras treated with NAC or vehicle. Mean ± SEM from 6 mice per group pooled from three independent experiments. (**e**–**g**) Phospho-p38 MFIs in different BM subsets. Mean ± SEM from 6 mice per group pooled from 3 independent experiments. (**h**,**i**) Percentages of Annexin-V^+^ cells in BM LSK and LK cells. Mean ± SEM from 8 mice per group pooled from 2 independent experiments. (**j**) Representative γH2AX staining (red) in BM LSK cells of *CXCR4*^+/+^ and *CXCR4*^−/−^ chimeras. Nuclei were stained with DAPI (blue). (**k**) Frequencies of γH2AX-positive BM LSK cells. More than 100 cells were counted per group in 3 independent experiments. Mean ± SEM from 3 independent experiments with 3 to 5 mice per group.

To assess whether high ROS levels were responsible for the observed functional deficiencies of CXCR4^−/−^ HSPCs, CXCR4^+/+^ and CXCR4^−/−^ chimeras were treated for one month with the permeant thiol N-acetyl-L-cysteine (NAC), which acts as an antioxidant agent. NAC treatment specifically diminished elevated ROS levels in LSK-SLAM, LSK and LK BM populations of CXCR4^−/−^ chimeras (Fig. 3a–c) but not in mature cells (Fig. 3d). The NAC-mediated decrease in ROS levels in CXCR4^−/−^ HSPCs correlated with an important decrease in p38 MAPK phosphorylation (Fig. 3e–g). No significant changes were observed in CXCR4^+/+^ HSPC populations. A high percentage of Annexin-V^+^ cells was detected in CXCR4^−/−^ LSK and LK populations compared to CXCR4^+/+^ counterparts. This substantial apoptosis was almost completely reverted by NAC treatment *in vivo* (Fig. 3h,i). Analysis of the level of γH2AX foci, a biomarker of DNA double-strand breaks revealed a significant increase in DNA damage in sorted CXCR4^−/−^ LSK cells compared to controls, and this phenomenon was entirely reverted by NAC treatment *in vivo* (Fig. 3j,k).

### *In vivo* treatment with the antioxidant NAC ameliorates the functional deficit of CXCR4^−/−^ HSPCs without influencing their proliferative state

**Figure 4:**
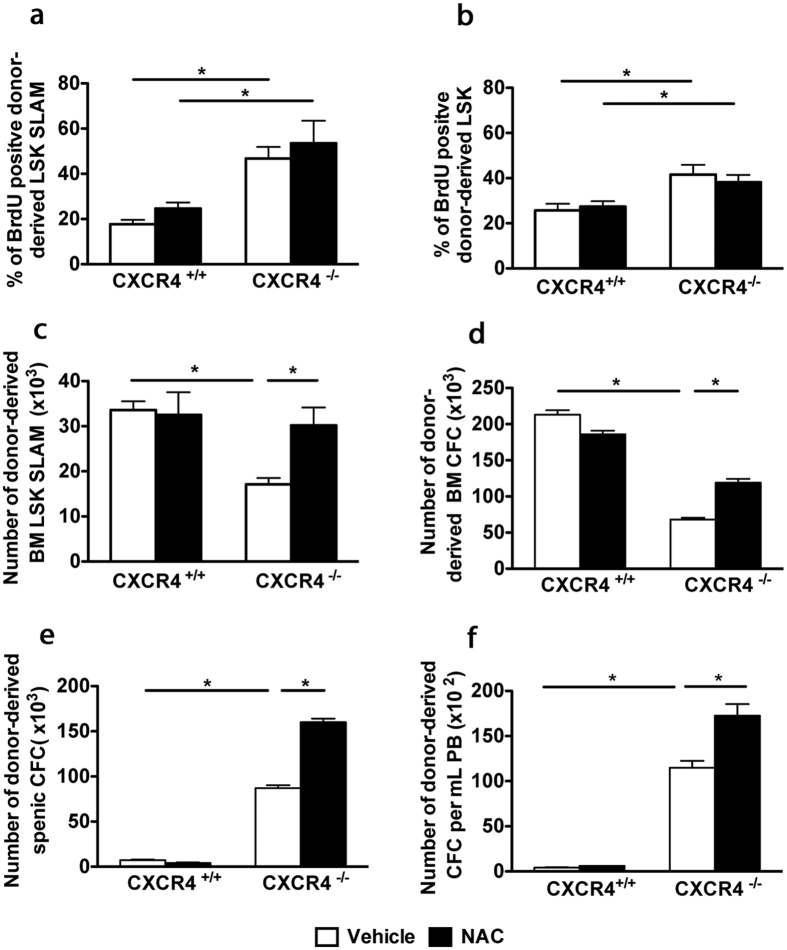
*In vivo* treatment with NAC ameliorates the functional deficit of CXCR4^−/−^ HSPCs without influencing their proliferative status. (**a**,**b**) CXCR4^+/+^ and CXCR4^−/−^ chimeras were treated with NAC or vehicle, BrdU^+^ cell frequencies in BM LSK-SLAM and LSK subsets. Data represent mean ± SEM from 6 mice per group pooled from 2 independent experiments. (**c**) Total number of BM LSK-SLAM cells. Data represent mean ± SEM from 8 mice per group, pooled from 2 independent experiments. (**d**–**f**) Restoration of total CFC numbers after NAC-treatment in BM, spleen and peripheral blood (PB). Mean ± SEM of colonies scored for 6 samples per group of 2 independent experiments performed in duplicate.

CXCR4 and CXCL12 have previously been shown to play a crucial role in regulating HSC quiescence, and their deficiencies had been associated with quiescence loss, correlating with decreased hematopoietic potential^28^,^29^. On the other hand, ROS were shown to drive HSCs into cell cycle^15^,^33^. To assess whether the quiescence loss of CXCR4^−/−^ HSCs was related to increased ROS levels, we treated CXCR4^+/+^ and CXCR4^−/−^ chimeras for one month with NAC and evaluated HSC proliferation *in vivo*. In baseline conditions, 48% of CXCR4^−/−^ LSK-SLAM cells had incorporated BrdU over a four-day-period, whereas only 18% of CXCR4^+/+^ LSK-SLAM cells were labeled with BrdU (Fig. 4a), supporting the idea that CXCR4^−/−^ HSPCs are cycling. Similar results were obtained with donor-derived LSK cells (Fig. 4b). However, after NAC treatment, CXCR4^−/−^ LSK-SLAM and LSK cells remained highly proliferative (Fig. 4a,b), indicating that elevated ROS levels are not the primary cause of the defect of CXCR4^−/−^ HSC quiescence.

We next asked whether NAC treatment could counteract the hematopoietic deficit observed in CXCR4^−/−^ chimeras. The total number of CXCR4^−/−^ LSK-SLAM cells was significantly higher after NAC treatment of CXCR4^−/−^ chimeras (Fig. 4c), whereas the same treatment did not affect CXCR4^+/+^ LSK-SLAM cells. A similar result was observed for CFC progenitors (Fig. 4d). Consistent with an increase in the BM CXCR4^−/−^ HSPC pool, a concomitant increase in circulating and splenic progenitors was also noticed upon NAC treatment (Fig. 4e,f). Thus, treatment of CXCR4^−/−^ chimeras with NAC could partially counteract the decrease in the number of BM HSPCs, indicating that elevated endogenous ROS contributes to CXCR4^−/−^ HSPCs defects.

### CXCR4/CXCL12 axis prevents buthionine sulfoximine-induced oxidative stress in HSPCs

**Figure 5:**
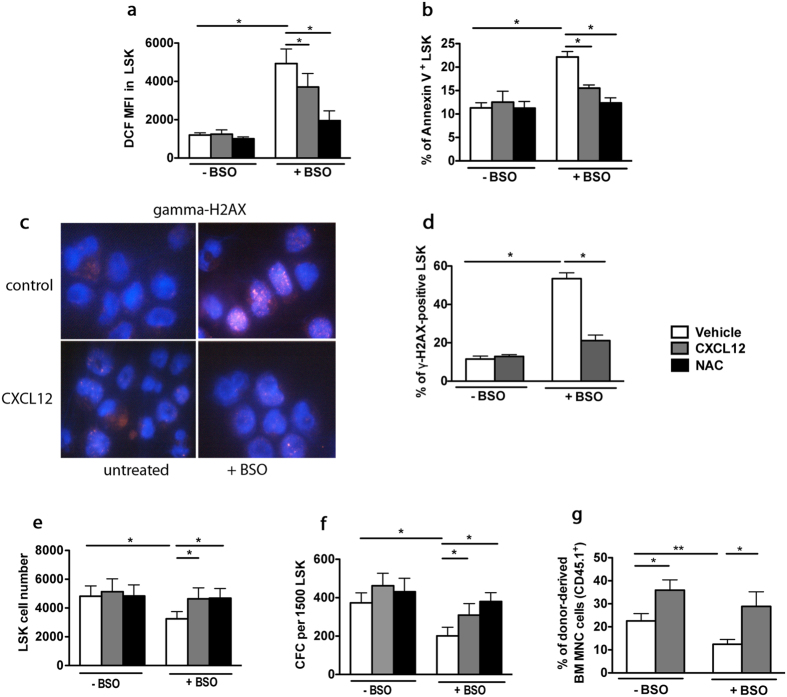
CXCR4/CXCL12 axis prevents BSO-induced oxidative stress in HSCs. (**a**) DCF MFIs of sorted WT LSK cells cultured for 48 hr with vehicle or BSO, in the absence or presence of 100 ng/mL CXCL12 or 2 mM NAC. Mean ± SEM, n = 6. (**b**) Annexin-V staining of LSK cells. Mean ± SEM, n = 3. (**c**) γ-H2AX-staining of LSK cells cultured for 48 hr in the absence (vehicle) or presence of BSO and CXCL12. Representative pictures are shown. (**d**) Percentages of γ-H2AX-positive LSK cells. More than 100 cells were counted per group in 3 independent experiments. (**e**) Effect of BSO-treatment on LSK proliferation. Mean ± SEM, n = 4. (**f**) Colony numbers (CFC) from LSK cells. Mean ± SEM of colonies from 5 independent experiments performed in duplicate. (**g**) CXCL12 rescues compromised hematopoietic long-term reconstitution with BSO-treated LSK cells. Before transplantation into lethally irradiated WT CD45.2^+^ recipients, CD45.1^+^ LSK cells were treated with BSO for 48 hr in the absence or presence of CXC12. Mean ± SEM of percentages of CD45.1^+^ donor-derived cells 6 months after transplantation from the pool (10 mice) independent experiments.

In the bone marrow stem cell niche, ROS regulation is complex. To test whether ROS regulation by CXCR4/CXCL12 signaling could be recapitulated *in vitro*, we used buthionine sulfoximine (BSO), a glutathione (GSH)-depleting agent. BSO was previously shown to promote oxidative stress in HSCs thus shortening their lifespan^12^,^34^,^35^. When sorted WT LSK cells were treated for 48 hr with different BSO concentrations, we detected a dose-dependent increase in ROS levels with a maximal effect at 100 μM (Supplementary Figure S2a). Addition of CXCL12 or NAC to the culture medium did not modify baseline ROS levels, but significantly decreased BSO-induced ROS (Fig. 5a). This effect was detected at CXCL12 concentrations as low as 2 ng/mL (Supplementary Figure S2b). As expected, BSO induced a sharp decrease in the GSH/GSSG ratio, indicating an important cellular depletion in glutathione (Supplementary Figure S2c). Of note, this decrease was still observed in the presence of CXCL12, indicating that the rescue effect of CXCL12 on ROS did not depend on the glutathione pathway (Supplementary Figure S2c). The increase in the percentage of apoptotic (Fig. 5b) and γH2AX foci-expressing LSK cells (Fig. 5c,d) observed in presence of BSO, were lowered by CXCL12 or NAC. In addition, CXCL12 and NAC rescued the deleterious effects of BSO on both, cell proliferation and colony-forming capacity of LSK cells (Fig. 5e,f, Supplementary Figure S2d, S2e).

Finally, we studied the effect of CXCL12 on the hematopoietic repopulation capacity of BSO-treated LSK cells, evaluated 6 months after transplantation. CXCL12 addition did slightly, yet significantly increase the hematopoietic reconstitution potential of LSK cells under baseline conditions. Even more, it remarkably counteracted the deleterious effects of BSO (Fig. 5g). The rescued reconstitution capacity of BSO-treated HSCs was confirmed by the detection of donor-derived B cells, T cells and myeloid cells in recipient mice (data not shown). Altogether, these results show that CXCR4/CXCL12 axis has an important role in the protection of HSCs against oxidative stress.

### CXCR4^−/−^ HSPCs cells exhibit increased sensitivity to oxidative stress induction

**Figure 6:**
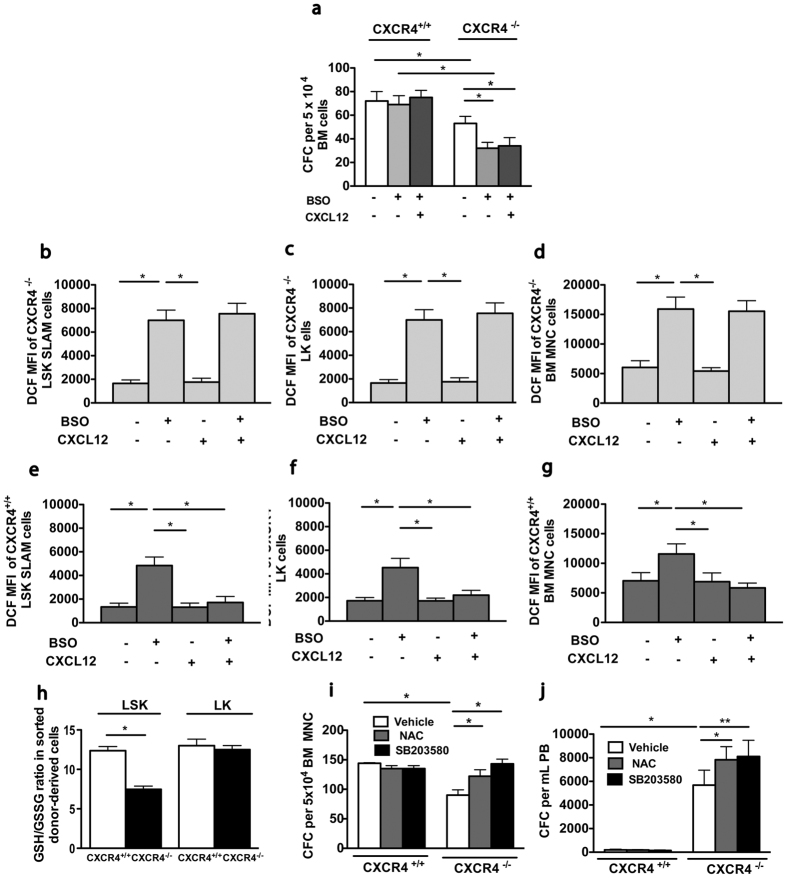
CXCR4^−/−^ HSPCs exhibit enhanced sensitivity to oxidative stress. Donor-derived BM cells from CXCR4^+/+^ and CXCR4^−/−^ chimeras 6 weeks post transplantation were sorted and cultured for 24 hr in the presence of a low BSO concentration (10 μM), with or without CXCL12. (**a**) Colony formation. Mean ± SEM of 3 independent experiments performed in duplicate. (**b**–**d**) DCF MFIs on CXCR4^−/−^ BM LSK-SLAM (**b**), LK (**c**) and BM-MNC cells (**d**). **(e–g**) DCF MFIs on CXCR4^+/+^ BM LSK-SLAM (**e**), LK (**f**) and BM-MNC cells (**g**). Mean ± SEM of 3 independent experiments, with 3 mice per group. (**h**) GSH/GSSG ratios in sorted LSK and LK cells from CXCR4^+/+^ and CXCR4^−/−^ chimeras, 6 weeks after transplantation. Mean ± SEM of 3 independent experiments performed in triplicate, with 4 mice per group. (**i**,**j**) CFC frequencies in sorted CD45.2+ BM and in peripheral blood cells from CXCR4^+/+^ and CXCR4^−/−^ chimeras determined 3 months after transplantation. Sorted cells were plated with vehicle, NAC (2 mM) or p38 MAPK inhibitor SB203580 (25 μM). Mean ± SEM of 3 independent experiments performed in duplicate.

As CXCR4^−/−^ HSPCs are characterized by elevated endogenous ROS levels compared to CXCR4^+/+^ counterparts, we explored the possibility that they could be particularly vulnerable to additional oxidative stress. Sorted CD45.2^+^ BM cells from ten-week-old CXCR4^+/+^ and CXCR4^−/−^ chimeras were treated *in vitro* for 24 hours with a suboptimal dose of BSO (10 μM) and then seeded in semi-solid culture. Strikingly, whereas this low concentration of BSO had no impact on CXCR4^+/+^ cells, it reduced the number of CXCR4^−/−^ progenitors by about 50% (Fig. 6a). Furthermore, ROS levels were increased in all CXCR4^−/−^ BM cell subsets, including LSK-SLAM, LK and BM mononuclear cells (Fig. 6b–d) to a higher degree than in their CXCR4^+/+^ counterparts (Fig. 6e–g). CXCL12 addition had no effect on basal ROS levels, but resulted in substantial reduction of ROS levels in BSO-treated CXCR4^+/+^ cells, but had not effect on CXCR4^−/−^ cells (Fig. 6b–g). As these results indicate that CXCR4^−/−^ progenitors are more amenable to oxidative stress induction, we interrogated whether glutathione, the major cellular antioxidant reservoir, was altered in CXCR4^−/−^ cells. As shown in Fig. 6h, the GSH/GSSG ratio was significantly lower in CXCR4^−/−^ LSK BM cells, compared to CXCR4^+/+^ counterparts. This was specific for CXCR4^−/−^ LSK cells, as the GSH/GSSG ratio was unchanged in CXCR4^−/−^ LK cells with respect to CXCR4^+/+^ LK cells. In line with a low antioxidant reservoir in CXCR4^−/−^ LSK cells, the expression of several genes encoding ROS-detoxifying enzymes, such as Prdx1, Txn2, Gpx3 (Supplementary Figure S3a) and the NRF2 nuclear targets, *Hmox1* and *Nqo1*, (Supplementary Figure S3b), were overexpressed in LSK cells of CXCR4^−/−^ chimeras compared to CXCR4^+/+^ chimeras. These results are in line with the notion that reactive intracellular defense mechanisms are frequently induced in response to a low cellular antioxidant status.

Recent studies have indicated that oxidative stress induced by culture in ambient air compromises the survival of mouse and human HSPCs^36^. We thus addressed the question, whether CXCR4 deficiency resulted in an increased sensitivity to *in vitro* culture. The number of clonogenic progenitors that had grown from CD45.2^+^ BM cells of CXCR4^−/−^ chimeras, was lower compared to CXCR4^+/+^ counterparts and NAC addition resulted in an increase in CXCR4^−/−^ progenitor cell numbers (Fig. 6i). Similarly, the number of progenitor cells that had grown from CXCR4^−/−^ PB also increased, when NAC was added to the culture medium (Fig. 6j). Furthermore, addition of the p38 MAPK inhibitor SB203580 increased BM and PB colony-forming capacity of CXCR4^−/−^ cells (Fig. 6i,j). Altogether, these results suggest that CXCR4^−/−^ HSPCs are more vulnerable to oxidative stress induced by *in vitro* culture and that activated p38 MAPK pathway is involved in this phenomenon.

### Mitochondria of HSPCs are targets for CXCL12 in oxidative stress control

**Figure 7:**
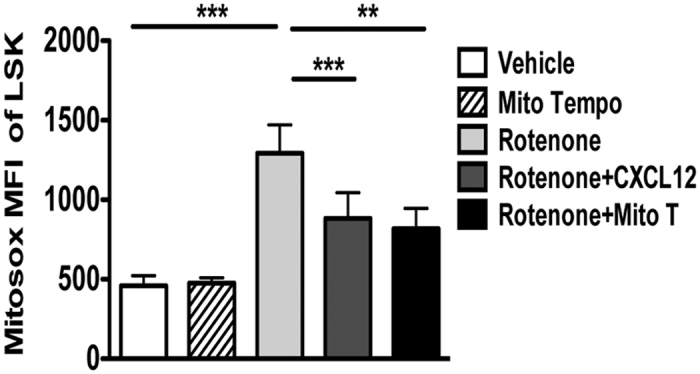
Mitochondria are targets for CXCL12 in oxidative stress control. CXCL12 or the mitochondrial ROS scavenger Mito Tempo reduces Mitosox Red MFI in sorted LSK cells. Cells were treated with rotenone (5 μM) or vehicle for 1 hour in absence or presence of CXCL12 (10 ng/ml) or Mito Tempo (2 μM). Mean ± SEM of 3–7 independent experiments.

Oxidative damage by ROS generated in mitochondria is a potent cause of HSC dysregulation^37^,^38^. We thus tested, whether CXCL12 could regulate mitochondrial oxidative stress, by treating WT LSK cells with rotenone, a specific inhibitor of the complex I of the mitochondrial electron transport chain^39^. Rotenone significantly increased mitochondrial ROS levels, as detected by MitoSOX Red, an effect that could be prevented by preincubation with the mitochondrial ROS scavenger Mito Tempo (Fig. 7). Strikingly, CXCL12 also lowered MitoSOX Red staining (Fig. 7), suggesting that CXCL12 can rescue ROS at the mitochondrial level.

## Discussion

Although the management of ROS elevation in HSCs was proposed to be crucial for the maintenance of lifelong hematopoiesis, little is known about the processes by which HSCs deal with oxidative stress situations. Here, we demonstrate for the first time that CXCR4/CXCL12 axis can limit oxidative stress injury in HSCs by reducing mitochondrial oxidative stress.

CXCR4 signaling influences many aspects of HSPC biology including migration, retention within stem cell niches, proliferation and quiescence. Nonetheless, its requirement for HSPC biology remains to be explored. Furthermore, significant discrepancies were reported on the effects of CXCR4 deletion regarding the long-term maintenance of the HSC pool, using inducible mouse models. Indeed, CXCR4 deletion achieved with poly(I)-poly(C)-inducible Cre-transgenic mice, resulted in sharp deleterious effects on HSCs, whereas CXCR4^−/−^ HSC numbers were maintained in tamoxifen-inducible Cre-transgenic mice^28^,^29^. These discrepancies may relate to effects of poly(I)-poly(C) or tamoxifen that may alter baseline HSPC numbers by inducing proliferation and eventually apoptosis even in the absence of any genetic modifications^40^,^41^.

To answer the question if and how the absence of CXCR4 leads to alterations in HSC long-term maintenance, we used a CXCR4^−/−^ mouse syngenic transplantation model with CXCR4^−/−^ FL cells. This model has the advantage to be constitutive and not to use inducers that may interact with HSPC biology. Using this model, we have previously shown that CXCR4^−/−^ HSPCs have a marked defect in radioprotection of lethally irradiated mice and demonstrated the crucial role of CXCR4 in the retention of HSPCs in the BM microenvironment of chimeric mice^26^. Here, we revealed a progressive depletion of LT-HSCs in the BM of chimeric CXCR4^−/−^ mice that correlates with an elevation of their endogenous ROS levels. Interestingly, no differences in ROS levels could be detected between CXCR4^+/+^ and CXCR4^−/−^ FL HSCs, suggesting that ROS regulation by CXCR4/CXCL12 axis is specific to adult HSCs or to the site of hematopoiesis (bone marrow versus FL). The importance of CXCR4 on ROS levels was demonstrated mostly using the transplantation model of CXCR4^−/−^ and CXCR4^+/+^ FL cells. This model offers an advantage over CXCR4 inhibitor administration in that CXCR4 inhibitor may also target non-hematopoietic cells within the microenvironment. Therefore, its effects cannot be attributed solely to the direct inhibition of CXCR4 on hematopoietic cells. In addition, the use of CXCR4 inhibitors is limited by their low stability. Importantly, despite these limitations, we have demonstrated an elevation in endogenous ROS levels in both, CXCR4^−/−^ transplanted HSCs and upon CXCR4 inhibitor administration. Thus, the observed increases of ROS levels in CXCR4^−/−^ HSPCs might not only reflect an altered cellular response to the stress inflicted by transplantation of FL CXCR4^−/−^ cells into irradiated mice. It will be of interest in future studies to establish, whether more stable CXCR4 inhibitor administration could also result in p38 MAPK phosphorylation and apoptosis.

Elevated ROS levels have been reported to limit the lifespan of HSCs in Atm^−/−^ 33 and Fox3a^−/−^ 42 mice. Indeed, we have demonstrated that *in vivo* administration of the antioxidant NAC to CXCR4^−/−^ chimeric mice partially rescued their progressive hematopoietic deficit. Since increased ROS levels in CXCR4^−/−^ HSPCs were associated with an activation of the p38 MAPK pathway, it is likely that this pathway is involved in the deleterious consequences on progenitor growth and hematopoietic reconstitution capacity. In line with this, we showed that *in vitro* addition of the p38 MAPK inhibitor SB203580 rescued the low biological activity of clonogenic progenitors and LSK-SLAM cells from CXCR4^−/−^ chimeras.

Strikingly, the substantial increase in proliferation of CXCR4^−/−^ HSCs was not reversed by *in vivo* NAC administration, although there was a significant NAC effect on the replenishment of the HSC pool, associated with decreased apoptosis and DNA damage. This positions ROS as upstream effectors of apoptosis, DNA damage and loss of long-term maintenance in CXCR4^−/−^ HSCs. This contrasts with Atm^−/−^ mice, in which cell cycle regulation of HSCs was shown to be downstream to ROS production^12^. Thus, absence of ROS effect on the cell cycle status of HSPCs is in line with data showing that CXCR4/CXCL12 axis directly acts on the cell cycle by regulation of p57^kip2^ 29. Importantly, the *in vivo* antioxidant treatment delayed the exhaustion of the CXCR4^−/−^ HSC pool without rescuing HSC quiescence, suggesting that increased ROS level is the primary determinant for HSC exhaustion, in some way independently of the cell cycle status and that proliferation by itself does not necessarily result in exhaustion of hematopoietic stem cell function.

Our experiments established that ROS production in HSPCs of CXCR4^−/−^ chimeras impacts their survival and long-term maintenance in a cell autonomous manner. This elevated ROS production may also impact in a paracrine manner normal hematopoiesis and the bone marrow microenvironment. In this regard, it will be interesting to investigate whether an abnormal microenvironment had developed in CXCR4^−/−^ chimeras and whether this may result in abnormalities in hematopoiesis. Of note, long-term hematopoietic reconstitution, in a competitive context between CXCR4^+/+^ and CXCR4^−/−^ HSPCs is dominated by normal hematopoiesis (even with an excess of CXCR4^−/−^), suggesting a small if any impact of ROS production by CXCR4^−/−^ cells on normal hematopoiesis^24^,^26^.

Excessive ROS production in CXCR4^−/−^ was associated with an upregulation of several ROS-detoxifying enzymes, that may reflect the challenged status of CXCR4^−/−^ cells in dealing with oxidative stress. Detoxification of ROS and repair of oxidatively-damaged proteins depend primarily on the availability of reduced glutathione, whose loss is an early hallmark of the progression of cell death in response to stress stimuli^43^. Of note, LSK cells from CXCR4^−/−^ chimeras demonstrated a low intracellular reservoir of reduced glutathione, indicating a reduced cellular redox-buffering ability. In line with this, CXCR4^−/−^ LSK cells showed an increased sensitivity to additional stress situations, such as those induced by *in vitro* culture.

Previous studies have demonstrated that global expression of CXCL12 transgene in mice caused an upregulation of mitochondrial mass, but low mitochondrial activity in LSK cells. However, these studies did not address the direct effect of CXCL12 on mitochondrial oxidative stress^44^. We addressed this point using *in vitro* studies and established that CXCL12 could indeed reduce mitochondrial ROS in murine HSPCs. Although the paucity of HSCs, particularly observed in CXCR4^−/−^ chimeras, challenged the analysis of mitochondrial ROS in CXCR4^−/−^ cells, these results indicate that CXCL12 could help in reducing ROS levels by lowering mitochondrial activity in murine HSPCs. The relationship between CXCL12 and the control of mitochondrial activity remains to be clarified.

Of note, CXCL12 protective effects against oxidative stress have been observed in culture systems containing cytokines that induce proliferation in LSK cells, demonstrating the importance of CXCR4/CXCL12 signaling in the regulation of intracellular ROS levels under proliferative conditions. This could be of particular relevance under physiological activation, such as bleeding or stress circumstances in which HSCs have to leave their hypoxic niches and to switch from dormancy to a metabolically active state^40^,^45^. For instance, the stress-induced mobilization of HSPCs by G-CSF is in part related to ROS elevation resulting from c-Met activity^11^. This mobilization is associated with CXCL12 underproduction and an increased apoptotic rate of mobilized cells compared to their steady-state BM counterparts^46^. Thus, it is possible and indeed likely, that CXCL12 treatment during mobilization will improve the recovery of HSPCs and prevent their apoptosis by inhibiting endogenous ROS production.

In conclusion, this study underscore the protective role of CXCR4/CXCL12 axis and environmental BM signals to limit oxidative stress and their active participation in the maintenance of the HSC pool. This study hence opens avenues of research for the association of CXCR4 antagonists with antioxidant agents to decrease deleterious ROS effects, which are associated with stress hematopoiesis such as mobilization, bleeding and transplantation.

## Methods

### Adoptive transfer of FL cells and hematopoietic reconstitution

Mice were bred and maintained under specific pathogen free conditions with acidified water (pH 5.3) at the Animal Core Facility of Gustave Roussy Institute (n° E-94-076-11). Animal experiments were approved by Ethical Committee C2EA-26: Comité d’éthique en expérimentation animale de l’IRCIV moc.liamg@62aeec officially registered by the French Ministry of Research. All experiments were performed in accordance with relevant guidelines and regulations and officially authorized by the French Ministry of Research (Permit number: 2012–23), as per Directive 2010/63 prescriptions and transposition into French law and regulations. The generation of CXCR4^−/−^ C57BL/6J-Ly5.2 mice and transplantation assays performed with FL cells from CXCR4^−/−^ and CXCR4^+/+^ embryos into lethally irradiated C57BL/6J-Ly5.1 mice were described previously^26^. Briefly, 5 × 10^6^ CXCR4^+/+^ or CXCR4^−/−^ FL cells were injected into eight-week-old lethally irradiated mice and analyzed 3, 8 and 64 weeks after engraftment. C57BL/6 J (Ly5.2 and Ly5.1) mice were purchased from Charles River and Harlan laboratories, respectively. Reconstituted chimeric mice (one month post engraftment) received 1 mg/mL N-acetyl-L-cysteine (NAC) in the drinking water during one month and were analyzed at the end of the treatment. Donor-specific anti-CD45.2 and recipient-specific anti-CD45.1 antibodies were used to discriminate donor-derived cells from recipient cells. For hematopoietic reconstitution assays, WT LSK cells from C57BL/6-Ly5.1 mice were cultured for 48 hr in the absence or presence of BSO and/or CXCL12 in DMEM medium supplemented with 10% FBS, recombinant mSCF (100 ng/mL) and TPO (100 ng/mL), all from PeproTech (Rocky Hill, NJ, USA). 3000 LSK cells per mouse were then injected into lethally irradiated C57BL/6J-Ly5.2 mice.

### Flow cytometric sorting and analysis

Cell suspensions from mouse BM and spleen were collected and stained with allophycocyanin (APC)-conjugated rat anti-mouse lineage (Lin) antibodies, including anti CD3, B220, Gr-1, Ter119 and CD41 antibodies. For sorting of LSK cells, Lin^−^ cells were stained with phycoerythrin-Cyanine 7 (PE-Cy7) conjugated anti-Sca-1 and peridin chorophyll protein-cyanine 5.5 (PerCP-Cy5.5) conjugated anti-c-Kit antibodies. For analysis of LSK-SLAM cells in BM, spleen and blood of CXCR4^+/+^ and CXCR4^−/−^ chimeras, cells were stained with Alexa Fluor 700 conjugated anti-CD45.2 antibody, APC-Cy7-conjugated anti-CD41, PE-Cy7 conjugated anti-Sca-1, PerCP-Cy5 conjugated anti-c-Kit, PE conjugated anti-CD150 and Pacific Blue conjugated anti-CD48 antibodies. In HSC proliferation assays *in vivo* (BrdU experiments), the same multi-marker subset was used, except that APC-conjugated antibody against CD48 was applied. All antibodies were either from BD Biosciences or Biolegend, San Diego, CA, USA. Hematopoietic populations were analyzed or sorted on FACS LSRII or FACSCanto II and on Influx cytometry (BD Biosciences, Mountain View, CA, USA), respectively.

### Microarray expression analysis

RNAs were extracted from sorted LSK cells from CXCR4^+/+^ and CXCR4^−/−^ chimeric mice, using the RNeasy Micro protocol (QIAGEN). cDNA labeling, hybridization and data analyses were performed using oligonucleotide microarrays (4 × 44 K mouse Agilent Technologies, Palo Alto, CA, USA). Data were extracted from scanned images using Feature Extraction software (Agilent) with default settings. Data from all hybridizations were analyzed with Rosetta Resolver software.

### Assessment of p38 MAPK phosphorylation by flow cytometry

BM cells from CXCR4^+/+^ and CXCR4^−/−^ chimeras were first submitted to immunofluorescent staining of cell surface antigens as described above to allow later gating on mature and immature cell populations. After fixation and pemeabilization with BD Cytofix/Cytoperm Buffer, cells were labeled with anti phospho-p38 MAPK rabbit antibody (Thr180/Tyr182) or rabbit specific IgG, followed by goat anti-rabbit Alexa Fluor 488 antibodies. Positive staining was determined by analysis on FACSCanto II.

### Intracellular and mitochondrial ROS detection

Intracellular ROS was monitored by flow cytometry using the redox-sensitive dye 5-(and-6)-chloromethyl-2′,7′dichlorodihydrofluorescin diacetate, acetyl ester (CM-H_2_DCF-DA). Cells were loaded with 2 μM DCFH_2_-DA for 20 min at 37 °C. For detection of mitochondrial super oxide anion (O_2_^.−^) production, cells were loaded with 2 μM Mitosox 30 min at 37 °C. To show specific mitochondrial ROS production, sorted LSK cells were incubated 20 min at 37 °C with 10 μM of the mitochondrial ROS scavenger MitoTempo before induction of mitochondrial ROS production.

### Oxidative stress induction

BM cells from CXCR4^+/+^ and CXCR4^−/−^ chimeras were cultured for 24 hr with BSO (10 μM) or vehicle (DMSO). Oxidative stress induction in LSK and LK cells was achieved with BSO (100 μM, 24 hr or 48 hr).

### DNA double-strand breaks assay

LSK cells from CXCR4^+/+^ and CXCR4^−/−^ chimeras or WT LSK cells were incubated with mouse anti-phospho-H2AX (Ser139) antibody followed by staining with ALEXA-546-conjugated anti-mouse antibody. Nuclei were stained with DAPI for microscopic examination. Cells were considered positive when they displayed more than four γH2AX foci per cell.

### Statistical Analysis

Results given as mean ± standard SEM were analyzed with the two-tailed unpaired Student’s t-test. Differences were considered significant when P-value was *p < 0.05; **p < 0.01 and ***p < 0.001.

Additional methods are listed in Supplemental Procedures.

## Additional Information

**How to cite this article**: Zhang, Y. *et al*. CXCR4/CXCL12 axis counteracts hematopoietic stem cell exhaustion through selective protection against oxidative stress. *Sci. Rep.* **6**, 37827; doi: 10.1038/srep37827 (2016).

**Publisher's note:** Springer Nature remains neutral with regard to jurisdictional claims in published maps and institutional affiliations.

## Supplementary Material

Supplemental Information
