# Supplementary material for: CXCR4/CXCL12 axis counteracts hematopoietic stem cell exhaustion through selective protection against oxidative stress
Source: Sci Rep. 2016 Nov 25;6:37827. doi: 10.1038/srep37827 (PMC5122894; doi:10.1038/srep37827)
Supplement: Supplemental Information [file srep37827-s1.pdf]

Supplementary Information

CXCR4/CXCL12 axis counteracts hematopoietic stem cell exhaustion through selective protection against oxidative stress

Yanyan Zhang[1,10], Mallorie Dépond[1,10], Liang He[1], Adlen Foudi[2], Edward Owusu Kwarteng[1], Evelyne Lauret[3], Isabelle Plo[1], Christophe Desterke[4], Philippe Dessen[5], Nobutaka Fujii[6], Paule Opolon[7], Olivier Herault[8,9], Eric Solary[1], William Vainchenker [1], Virginie Joulin[1], Fawzia Louache[1,9,11*], Monika Wittner[1,9,11]

[1]Paris-Saclay University, UMRS-1170, Gustave Roussy,Villejuif, France

[2]Paris-Saclay University, INSERM U935, Andre Lwoff Institute, Paul Brousse Hospital, Villejuif

[3] Paris Descartes University, CNRS (UMR 8104), Inserm U1016, Institut Cochin, Paris,

[4]Paris-Saclay University, UFR Medicine, INSERM UMS 33, Andre Lwoff Institute, Paul Brousse Hospital, Villejuif.

[5]Bioinformatic platform, UMS AMMICA, INSERM US23/CNRS UMS3665, Gustave Roussy,Villejuif

[6] Kyoto University, Graduate School of Pharmaceutical Sciences, Kyoto, Japan

[7]Laboratoire de pathologie expérimentale, Gustave Roussy,Villejuif

[8]CNRS UMR 7292 GICC, Tours, France

[9]CNRS GDR 3697 MicroNiT

[10]These two authors contributed equally

[11]Co-senior author

Supplemental procedures

Antibodies, hematopoietic reconstitution and flow cytometric analysis. The following antibodies were used: anti-phospho-p38 MAPK rabbit mAb (Thr180/Tyr182, Cell Signaling Technology, Danvers, MA, USA); mouse monoclonal anti-phospho-H2AX (Ser139) antibody (Upstate Biotechnology, Millipore); Alexa Fluor 488 F(ab)$_2$ fragment of goat anti-mouse IgG antibody (Invitrogen, Carlsbad, CA, USA) ; Alexa Fluor 488 goat anti-rabbit IgG antibody (Invitrogen, Carlsbad, CA, USA); Alexa Fluor 546 goat anti-mouse IgG antibody (Invitrogen, Carlsbad, CA, USA); APC-Cy7-anti-TER-119, B220, CD3e, GR-1, PerCP-Cy5.5-anti-c-Kit, PE-Cy7-anti-Sca-1, PE-anti-CD150, APC-anti-CD48 and Alexa Fluor 700-anti-CD45.2 (Biolegend, San Diego, CA, USA). For determination of chimerism, PE-anti-CD45.1 and APC-anti-CD45.2 were used (Biolegend, San Diego, CA, USA). Hematopoietic populations were analyzed on FACS LSRII or FACS CANTO II and sorted on Influx cytometers (BD Biosciences).

Chemicals. γ-glutamyl-cysteine-synthetase inhibitor buthionine sulfoximine (BSO), the antioxidant N-acetyl-L-cysteine (NAC), Rotenone and DAPI were purchased from Sigma–Aldrich, St Louis, MO, USA. p38 MAPK inhibitor SB203580 was obtained from TOCRIS Bioscience, Bristol, UK. The BrdU Flow KIT was purchased from BD Biosciences, CA, USA, the Annexin-V Assay kit from BD Pharmingen, Franklin Lakes, NJ, USA. The fluorescent probes 5-(and-6)-chloromethyl-2',7'dichlorodihydrofluorescin diacetate, acetyl ester (CM-H_2DCF-DA), MitoSOX Red and MitoTempo were purchased from Invitrogen, Carlsbad, CA, USA. *CXCR4* inhibitor TN14033 (TN140) was previously described [47].

Histology. BM sections were fixed with formaldehyde, stained with hematoxylin/eosin/safran and examined by a Zeiss Axiophot microscope.

***In vitro* Colony-forming Cell assay.** Colony-forming cell (CFC) potential was assayed in semisolid methylcellulose medium (MethoCult M3234, Stem Cell Technologies, Vancouver, Canada) supplemented with recombinant cytokines, as described previously [26]. Colony-forming unit cells (CFU-C) were scored in duplicate seven days after plating.

Annexin-V Assay. Apoptosis was assayed by staining freshly harvested BM cells with lineage, stem and progenitor markers, followed by Annexin-V and DAPI staining according to the manufacturer's recommendations. Positive staining was determined by analysis on

FACS CANTOII (BD Biosciences, Mountain View, CA, USA).

GSH/GSSG-Glo Assay. GSH/GSSG ratio was determined in sorted CD45.2$^+$ donor-derived LSK and LK cells from CXCR4$^{+/+}$ and CXCR4$^{-/-}$ chimeras, 8 weeks after transplantation of FL cells. The GSH/GSSG-Glo assay kit (Promega, Madison, WI, USA), a luminescence-based system for the detection and quantification of total glutathione (GSH+GSSG), GSSG and GSH/GSSG ratios was used, according to the manufacturer's recommendations.

Retroviral Transduction. Retroviral gene transduction of cDNAs encoding mouse CXCR4 was performed with the retroviral vector Migr/IRES/EGFP. Three months after transplantation, CD45.2$^+$/Lin$^-$ cells were sorted from *CXCR4*$^{+/+}$ or *CXCR4*$^{-/-}$ chimeras and incubated for 24 hr with 100 ng/mL SCF and 100 ng/mL thrombopoietin, followed by incubation for 48 hr with retrovirus. The efficiency of gene transduction was evaluated by expression of EGFP. Transduced cells were transplanted into lethally irradiated Ly5.1 mice.

Expression of antioxidative genes. Analyses were performed by real-time qRT-PCR on the LightCycler® 480 microwell plate-based cycler platform (Roche Applied Science) using Universal ProbeLibrary assays designed with the ProbeFinder software (Roche Applied Science). Three micrograms of total RNA from each sample were reverse transcribed using the SuperScript® VILOTM cDNA Synthesis kit (Invitrogen) according to the manufacturer's instructions. Primers were purchased from Invitrogen and Universal ProbeLibrary probes from Roche Applied Science (nucleotide sequences of the primers and probes on request). All targets were concomitantly analyzed. Real-time qRT-PCR reactions were carried out in a total volume of 10 µL on 20 ng of cDNA using LightCycler® 480 Probes Master (Roche Applied Science). The LightCycler® 480 was programmed to an initial denaturation (95°C, 10 min) following by 45 cycles of 10 sec at 95°C, 30 sec at 60°C, 1 sec at 72°C and a final cooling step at 40°C for 10 sec. All reactions were run in triplicate, and average values were used for quantification. The antioxidant gene expression was determined comparatively to the housekeeping genes (GAPDH, TBP, ACTB). The relative antioxidant gene expression was analyzed using the 2exp(-ΔΔC(T)) method (Livak KJ and Schmittgen TD, Methods 2001;25:402-408).

HSC proliferation analysis. Two months after transplantation of FL cells, CXCR4$^{+/+}$ and CXCR4$^{-/-}$ chimeric mice were injected with BrdU (1 mg BrdU/kg body weight, i.p.). After 18 hr, BM cells were recovered and stained with lineage, stem and progenitor markers and

permeabilized, followed by staining with FITC-conjugated anti-BrdU according to the manufacturer's recommendations.

***In vivo* inhibitor studies.** *CXCR4* inhibitor TN14033 (TN140, 24 mg/kg/day) was administered by Alzet osmotic pumps for seven days (Durect Corporation, Cupertino, CA, USA) as previously described [48]. Control animals received pumps containing PBS.

Supplementary Figure S1

Progressive hematopoietic long-term deficit, increased extra medullary hematopoiesis and peripheral circulation of HSPCs in CXCR4$^{-/-}$ chimeras. (a,b) Total numbers of donor-derived colonies (CFCs) formed from CXCR4$^{+/+}$ and CXCR4$^{-/-}$ BM (2 femurs + 2 tibia) and spleen in colony-forming cell assay, evaluated 3 weeks, 8 weeks and 64 weeks after transplantation of CXCR4$^{+/+}$ and CXCR4$^{-/-}$ FL cells**.** BM and splenic CD45.2^{+} cells were sorted and plated in duplicate in semisolid medium under standard conditions (CXCR4$^{+/+}$ cells) or in the presence of 600 μg/mL G418 (CXCR4$^{-/-}$ cells). As CXCR4$^{-/-}$ FL cells carry a neomycin insertion cassette, G418 allows to validate for the origin of CFCs. Data are represented as mean ± SEM of colonies from CXCR4$^{+/+}$ and CXCR4$^{-/-}$ BM, spleen and PB samples obtained from 4-7 mice per group in 2 independent experiments. **(c-e)** Total numbers of donor-derived CD45.2^{+} LSK (spleen, PB) and LSK-SLAM (PB) cells of CXCR4$^{+/+}$ and CXCR4$^{-/-}$ chimeras. LSK-SLAM and LSK cell numbers were determined by immuno phenotyping, considering cellularity and chimerism of the respective organs. Data are represented as mean ± SEM of total cell numbers obtained from 4-7 mice per group in 2 independent experiments.

Supplementary Figure S2
Increased ROS levels after BSO treatments abrogate colony-forming capacity of HSPCs: rescue effect of CXCL12. (a) BSO-treatment *in vitro* for 48 hours increased ROS levels in sorted WT LSK cells from ten-week-old mice in a dose-dependent manner. ROS levels were significantly decreased when 10 ng/mL CXCL12 was simultaneously added to the culture medium. Data are mean ± SEM of DCF MFIs obtained in 5 independent experiments. **(b)** Dose-dependent rescue effects of CXCL12 on BSO-induced ROS level increases in LSK cells. Data are mean ± SEM of DCF MFIs measured in 5 independent experiments. **(c)** Freshly isolated WT LSK cells were cultured for 48 hours in the absence or presence of BSO alone or with CXCL12. The GSH/GSSG ratio was determined with the GSH/GSSG-Glo Assay. Data represent mean ± SEM of GSH/GSSG ratios from 3 independent experiments, performed in triplicate. **(d)** Dose-dependent inhibition of progenitor growth of sorted LSK cells by BSO in the absence or presence of 10 ng/mL CXCL12. Cells were cultured for 48 hr in the presence of BSO or vehicle, washed and plated in semisolid medium in duplicate. Data represent mean ± SEM from colony numbers of 5 independent experiments. **(e)** Dose-dependent rescue effects of CXCL12 on BSO-induced inhibition of LSK CFC potential. Data represent mean ± SEM from colony numbers of 3 independent experiments.

Supplementary Figure S3
ROS-induced up regulation of antioxidant enzyme expression in HSPCs of ten-week-old *CXCR4$^{+/+}$* and *CXCR4$^{-/-}$* chimeras. (a) Results of qRT-PCR assays for expression of antioxidant enzymes performed on donor-derived sorted LK (grey line) and LSK cells (black line) are presented as *CXCR4$^{-/-}$* over *CXCR4$^{+/+}$* expression ratios (mean +/- SD, n=3). Delta Ct values (vs Rpl13a) shown below indicate expression level of each transcript in LSK cells (bottom). **(b)** Results of qRT-PCR assays for expression of nuclear targets of NRF2 performed on donor-derived sorted LK (grey line) and LSK cells (black line) are presented as *CXCR4$^{-/-}$* over *CXCR4$^{+/+}$* expression ratios (mean +/- SD, n=3). Delta Ct values (vs Rpl13a) shown below indicate expression level of each transcript in LSK cells (bottom).

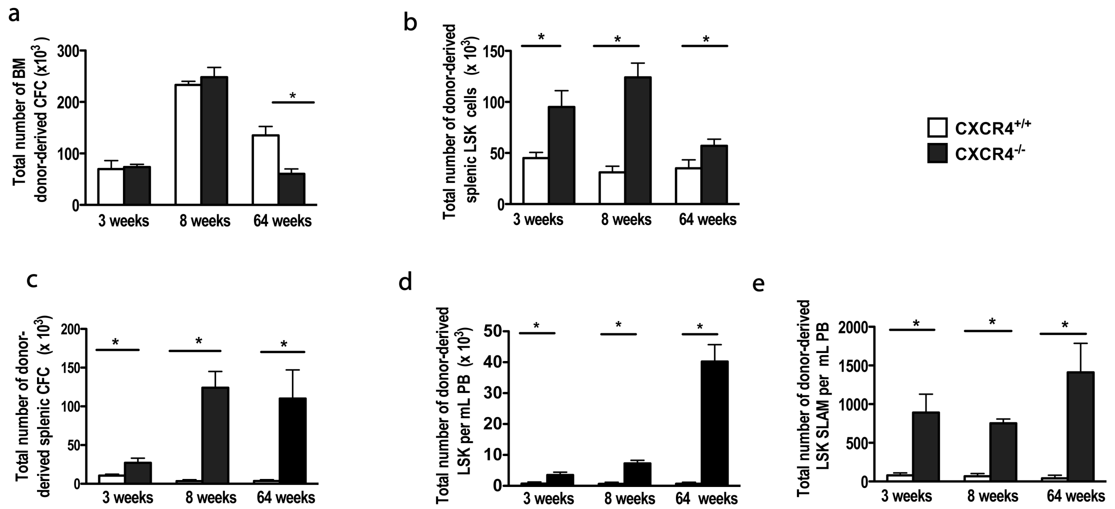

Supplementary Figure 1

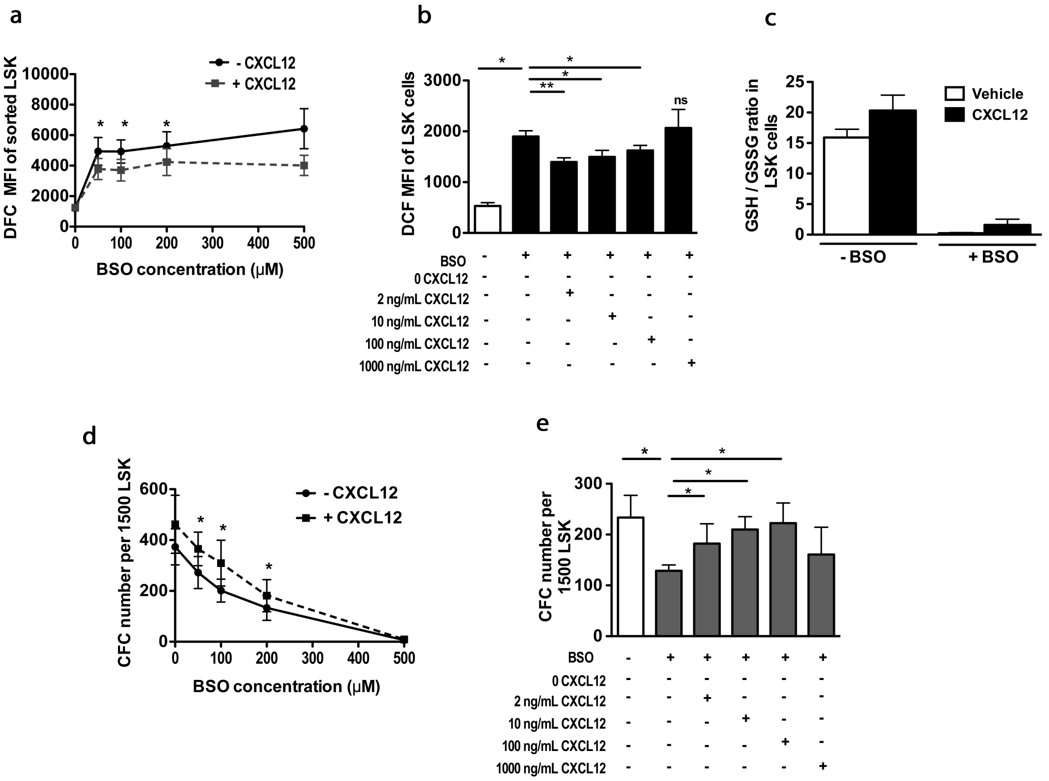

Supplementary Figure 2

a

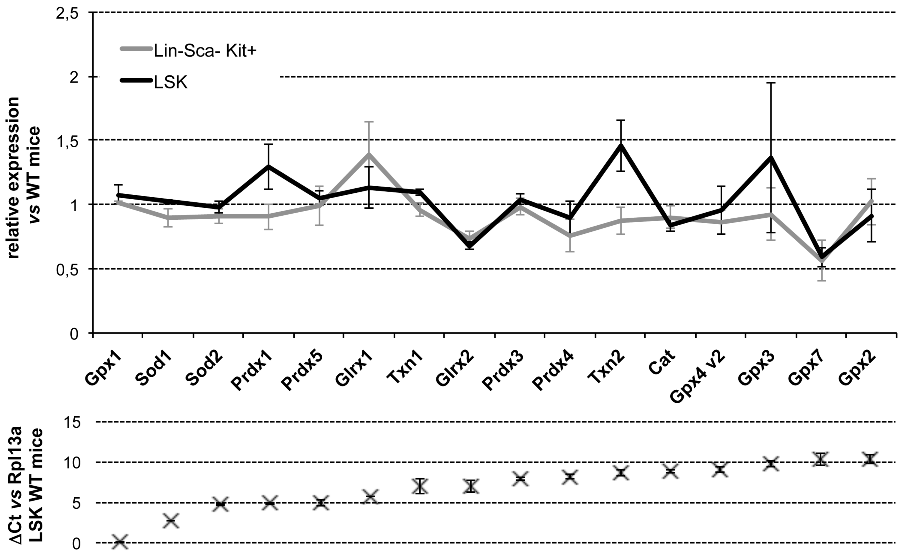

b

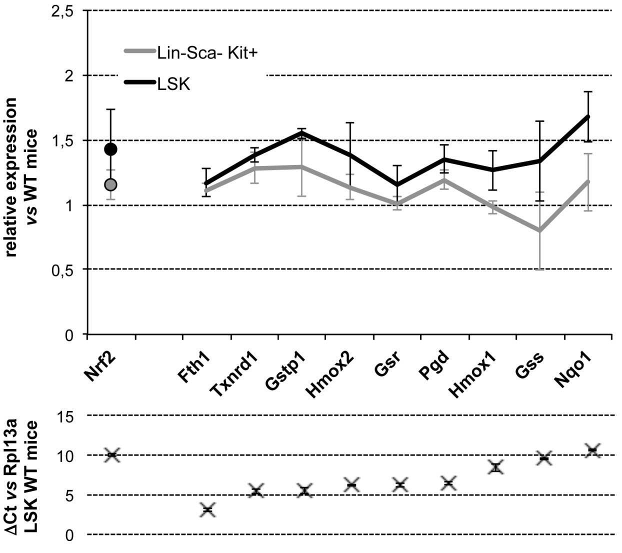

Supplementary Figure 3
